# Supplementary figures and images for: GSMN-ML- a genome scale metabolic network reconstruction of the obligate human pathogen Mycobacterium leprae
Source: PLoS Negl Trop Dis. 2020 Jul 6;14(7):e0007871. doi: 10.1371/journal.pntd.0007871 (PMC7365477; doi:10.1371/journal.pntd.0007871)

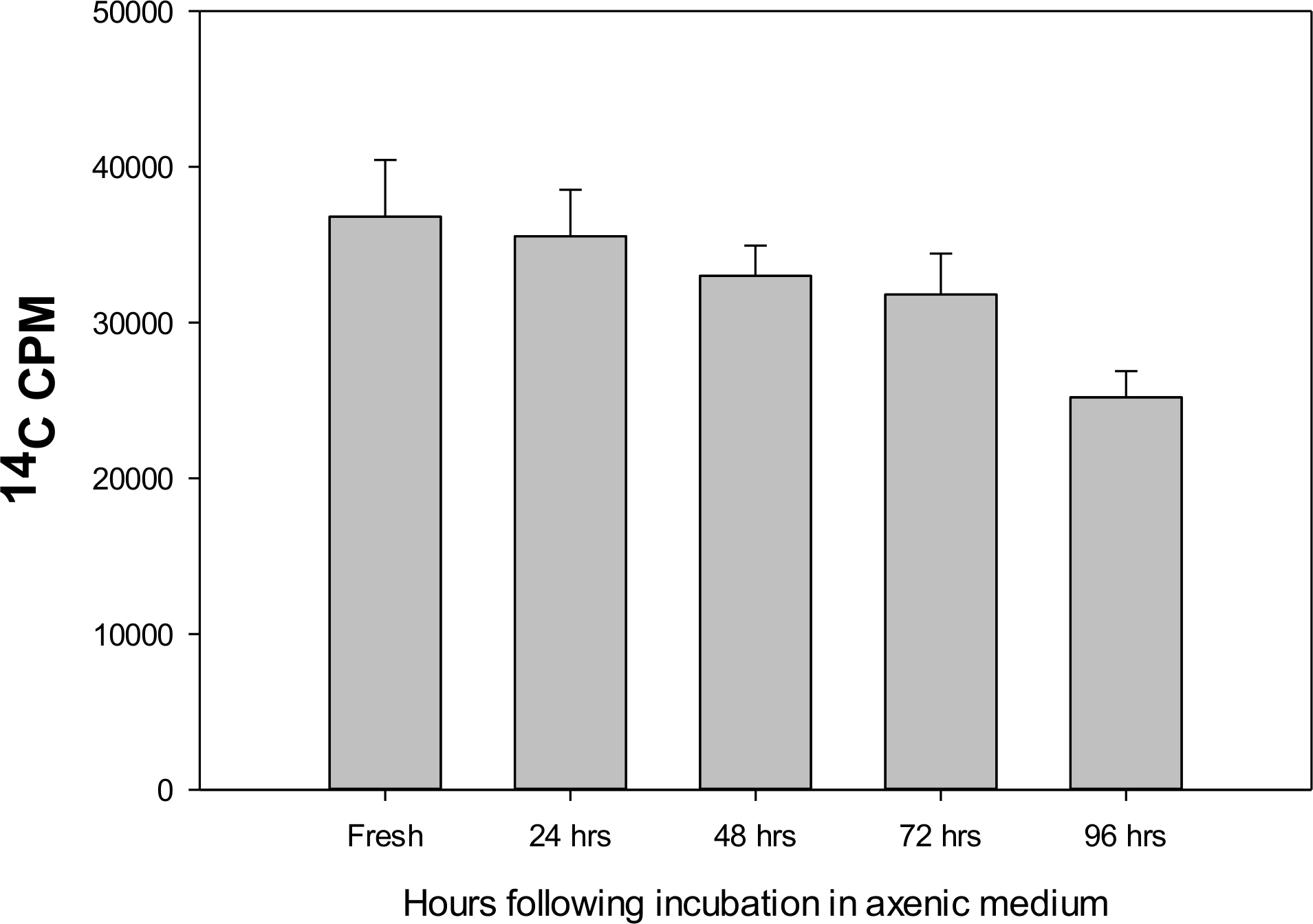

Supplement: S1 Fig — Radiorespirometry (RR) was used to measure oxidation rate of M. leprae using palmitic acid as the only carbon source [52]. For the RNASeq experiment the RR data shows that there was no significant difference (P = 0.209) in RR between fresh and following 48hrs incubation in the axenic medium, while after 96hrs of incubation there was a significant difference (P = 0.018). (TIF) [file pntd.0007871.s009.tif]
